# Worries about inadequate medical treatment in case of a COVID-19 infection: the role of social inequalities, COVID-19 prevalence and healthcare infrastructure

**DOI:** 10.1186/s12889-022-14024-9

**Published:** 2022-09-16

**Authors:** Alina Schmitz, Claudius Garten, Simon Kühne, Martina Brandt

**Affiliations:** 1 TU Dortmund University, Faculty of Social Sciences, Emil-Figge-Str. 50, 44227 Dortmund, Germany; 2 Bielefeld University, Faculty of Sociology, Universitätsstr. 25, 33615 Bielefeld, Germany

**Keywords:** Mental health, Population survey, Social and political issues, Health service research

## Abstract

**Background:**

This study investigates individual and regional determinants of worries about inadequate medical treatment in case of a COVID-19 infection, an important indicator of mental wellbeing in pandemic times as it potentially affects the compliance with mitigation measures and the willingness to get vaccinated. The analyses shed light on the following questions: Are there social inequalities in worries about inadequate medical treatment in case of a COVID-19 infection? What is the role of the regional spread of COVID-19 infections and regional healthcare capacities?

**Methods:**

Based on data derived from the *German Socioeconomic Panel* (SOEP), a representative sample of the German population aged 18 years and over, we estimated multilevel logistic regression models with individual-level (level 1) and regional-level (level 2) variables. The regional variables of interest were (a) the number of COVID-19 infections, (b) the number of hospital beds as an overall measure of the regional healthcare capacities, and (c) the number of free intensive care units as a measure of the actual capacities for treating patients with severe courses of COVID-19.

**Results:**

Women, older respondents, persons with migrant background and those with a lower socioeconomic status were more likely to report worries about inadequate medical treatment in case of a COVID-19 infection. Moreover, respondents with chronic illness, lower subjective health and those who consider COVID-19 as a threat for their own health were more likely to report worries. In addition, also regional characteristics were relevant. Worries were more common in poorer regions with higher COVID-19 infections and worse health infrastructure as indicated by the number of hospital beds.

**Conclusions:**

The analysis not only indicates that several social groups are more concerned about inadequate medical treatment in case of a COVID-19 infection, but also highlights the need for considering regional-level influences, such as the spread of the virus, poverty rates and healthcare infrastructure, when analyzing the social and health-related consequences of the pandemic.

## Background

The COVID-19 pandemic and the related mitigation measures have led to worldwide decreases in mental wellbeing, as measured by anxiety, depressive symptoms and loneliness [1, 2], as well as to an increase of COVID-19 related fears [3, 4]. The pathways through which the pandemic can affect mental wellbeing are manifold, including changes in daily routines and reduced social contacts [5, 6], as well as economic hardship due to short-time work or job loss [7, 8]. Moreover, people face the risk of a potentially harmful and even life threatening COVID-19 infection, which can trigger feelings of fear and distress and which can even lead to individuals’ withdrawal from social activities [9, 10]. Especially being exposed to the risk of infection can increase fear and distress [11].

In many countries all over the world, the COVID-19 pandemic has pushed healthcare systems to the brink of collapse. Not only in countries with few economic resources [12], but worldwide sharp increases in the number of patients with severe courses of COVID-19 have overburdened hospital systems and negatively affected health care. Some facilities had to adopt a “crisis standard of care” due to resource limitations with respect to available hospital space, staffing and medical supplies. As the health professionals’ focus shifted to addressing the immediate care needs of a high number of COVID-19 patients, many preventive and elective procedures were suspended [13]. As the capacities of intensive care beds and medical equipment were exceeded in some regions, healthcare professionals even had to prioritize COVID-19-patients who require therapy more urgently. In times of absolute scarcity of intensive care beds, medical doctors had to make “life and death ‘triage’ decisions about who has access to needed treatments” [14].

Against this background, this study investigates the role of individual and regional social inequalities, the regional spread of COVID-19 and healthcare infrastructure for worries about inadequate medical treatment in case of a COVID-19 infection in Germany. Although Germany has a well-equipped health system, the need for triage was also discussed here [15]. Apart from representing a threat for mental wellbeing [16] worries about inadequate medical treatment might influence individuals’ compliance with mitigation measures and the willingness to get vaccinated [4, 17] and thus are a highly relevant topic for public health. This study sheds light on the following questions: Are there social inequalities in worries about inadequate medical treatment in case of a COVID-19 infection? What is the role of the regional spread of COVID-19 infections and healthcare capacities?

### Social inequalities in mental wellbeing in light of the pandemic

Since the outbreak of the COVID-19 pandemic, social inequalities in mental wellbeing have been documented for a variety of characteristics. Not only measures of physical distancing and regulations of quarantine, but also the fear of infection or contraction have affected mental wellbeing [18, 19]. Female gender, younger age, a lower educational level, unemployment, being single and living alone were associated with several indicators for mental wellbeing, including depressive symptoms, anxiety, fearfulness and loneliness [1, 2, 20] Inequalities in mental wellbeing are also evident with regard to worries specifically related to COVID-19. While women’s concerns revolve around the impact of the pandemic on their loved ones and severe health consequences, men are more concerned about the impact on the economy and society as a whole [21, 22]. Moreover, COVID-19-related strains in mental wellbeing differ by physical health status, as persons with chronic health conditions and current or past psychiatric treatment report higher levels of distress relative to their counterparts without health limitations [1, 23]. Although older age has been identified as a risk factor for severe illness in case of a COVID-19 infection, there is evidence that older age is associated with lower levels of anxiety and depression during the pandemic [7, 24, 25].

With regard to socioeconomic status (SES), a well-known determinant of mental wellbeing in non-pandemic times (e.g. [26]), studies provide inconclusive results. Some studies show higher levels of depression, anxiety and psychosocial distress in individuals with low educational level [1], in persons with low income [27, 28] and especially in those who experience COVID-19-related income loss [29]. Other studies, in contrast, find no evidence for socioeconomic inequalities in mental wellbeing – at least at the beginning of the pandemic [23]. Knowledge about COVID-19 has been identified as a factor that prevents negative outcomes on mental health [30–32].

### Contextual influences and the role of healthcare infrastructure

Previous research has shown that besides individual characteristics, also contextual factors are relevant for mental wellbeing, but that also contextual factors play a role. In pre-COVID times, mental disorders [33] and especially anxiety and depressive symptoms [34–36] in urban compared to rural areas. In addition, mental health problems were more prevalent in economically deprived neighbourhoods and neighbourhoods with low levels of social cohesion [37, 38]. Moreover, some studies suggest that the regional infrastructure is associated to mental wellbeing. Davern and colleagues [39] show that the accessibility of social infrastructure (including community centres, culture and leisure, childcare services, schools, education, health and social services, as well as sports and recreation) was associated with higher wellbeing. In addition, healthcare infrastructure seems to be important for wellbeing – at least in the older population for which Verbakel [40] and Wagner and Brandt [41] showed that the national respective regional availability of long-term care services is associated to the wellbeing of informal caregivers.

Regarding COVID-19-related strains in wellbeing, research on contextual determinants is very limited. Cross-national comparative studies documented an increase of fear with rising COVID-19 cases [3, 4]. However, evidence on intra-national inequalities on the regional level is inconclusive [42–44]. Moreover, there are a few studies on rural-urban differences that show greater fear of COVID-19 infection and more loneliness in urban and more densely populated areas while generalized anxiety was more prevalent in less densely populated areas [45, 46]. Other regional aspects potentially related to fear of COVID-19, such as healthcare infrastructure, have not been investigated yet, although health care accessibility has been shown to be related to COVID-19 mortality on a regional level [47, 48].

### Objectives and design of this study

Based on multilevel models using cross-sectional data from the *German Socioeconomic Panel Study* (SOEP), this observational study analyses social and regional inequalities in worries about inadequate COVID-19-treatment in the German population aged 18 years and older. In contrast to most of the existing studies that rely on convenience samples, our analyses are based on a representative sample of the adult German population. While a few previous studies have investigated the role of socio-demographic and health-related factors for mental wellbeing in light of the pandemic, no study has investigated the role of regional contextual factors. We include several indicators of regional characteristics: the prevalence of COVID-19 infections, population density, healthcare infrastructure and economic resources. Furthermore, our study complements previous research by analysing worries about inadequate medical treatment – an indicator for COVID-19 related strains in mental wellbeing that has not been investigated before, but which is relevant for public health research and policy makers as it potentially affects the compliance with mitigation measures as well as the willingness to be vaccinated against COVID-19.

## Methods

### Data

We use data from the SOEP, a multidisciplinary panel study with representative samples of private households in Germany. Nearly 15,000 households and about 30,000 persons participate in the regular survey. Core topics of the questionnaire include education, occupation and employment, housing, physical and mental health, as well as attitudes, values and personality characteristics (see [49]). In April 2020, the project “SOEP-CoV – The Spread of the Coronavirus in Germany: Socio-Economic Factors and Consequences” was initiated as a collaboration of SOEP and Bielefeld University in Germany (funded by the Federal Ministry of Education and Research, BMBF). A subsample of the regular SOEP-sample was interviewed in two waves of telephone surveys, one wave from April to July 2020 and another one from January to February 2021 [50].

For our analysis, we selected data from the second SOEP-CoV wave from January and February 2021, when COVID-19 infections in Germany varied between 57 and 167 infections within the last 7 days per 100.000 inhabitants [51, 52]. We did not include the data from the first wave, as COVID-19 incidences were very low at that time and there were no shortages in medical care.

We match the micro-data from SOEP-CoV with macro-data on the regional level using the NUTS (Nomenclature of Territorial Units for Statistics) classification. This classification divides the territory of the European Union into hierarchical systems in order to enable statistical comparisons at various regional levels. The three hierarchical levels are known as NUTS-1, NUTS-2 and NUTS-3 [53]. Our analysis focusses on the 38 German NUTS-2 regions (governmental districts, “Regierungsbezirke”, former “Regierungsbezirke and Länder”) where population sizes ranged from 533,133 to 5,207,457 inhabitants on January 1st 2020 [54]. The regional data was retrieved from the Regionaldatenbank Deutschland by the Federal Statistical Office and the statistical offices of the Länder [54] and *infas 360 GmbH* [55, 56]. The data from *infas 360 GmbH* is based on two sources: the *Robert-Koch-Institut* (RKI), which is the Germany’s central scientific institution in the field of biomedicine [57] and the *Deutsche Interdisziplinäre Vereinigung für Intensiv- und Notfallmedizin* (DIVI).

The SOEP-sample for our analysis was restricted to respondents for whom data linkage with regional information was possible. Furthermore, we excluded respondents with missing information on one of the variables of interest on the micro-level while all indicators were completely available for the NUTS-2 regions. This reduced the initial sample from 6013 to 5045 respondents from all 38 German NUTS-2 regions. The imputation of missing values did not alter the regression results substantially, thus, only complete case analysis results will be presented. On average, regions include 133 respondents with a minimum of 23 and a maximum of 340 respondents (median (*p*50) = 100, *p*5 = 42, *p*95 = 326). For multilevel models, a threshold of 30 observations per level to estimated contextual effects has been proposed. We performed the analysis with and without regions with less than 30 inhabitants [58]. As the results did not differ, analyses with the full sample will be presented in the following.

### Variables

Our outcome of interest, worries about inadequate COVID-19 treatment, was measured by the question: “How concerned are you about the following? About whether you will receive the necessary medical treatment if you contract the coronavirus” with answer categories “very concerned”, “somewhat concerned” “not concerned at all”. For our analysis, we created a binary variable with 0 = “not concerned at all” and 1 = “somewhat concerned” or “very concerned”. This question was only directed to respondents who had not suffered a COVID-19 infection at the time of the interview.

As predictors on the individual level, we considered variables that have been identified as important determinants of mental wellbeing in previous studies: gender, age, migration background, educational attainment (as measured by the CASMIN-classification) and household net equivalence income. Furthermore, we considered several health-related indicators: the presence of at least one chronic disease (diabetes, asthma, heart disease, cancer, stroke or high blood pressure), as well as the respondent’s subjective evaluation of his/her health status. Moreover, we considered if the respondent expected to have a life-threatening course of the disease in case of a COVID-19 infection as a measure whether the respondents consider the virus as a relevant risk for his/her own health. We also included the type of health insurance. Around 88% of the German population have a statutory health insurance, while around 11 % of the population have a private health insurance [59]. In Germany, the statutory health insurance provides access to high-quality medical treatment, but private health insurance usually covers a much wider range of medical treatments.

As predictors at the regional level of NUTS-2 regions, we included the number of COVID-19 infections per 1000 inhabitants throughout the period of the SOEP-data collection. Regarding healthcare infrastructure, we included two measures: Firstly, the number of hospital beds per 1000 inhabitants in 2019 as an overall measure of the regional healthcare capacities and secondly, the average number of free intensive care units per 100,000 inhabitants throughout the period of SOEP-data collection. The latter is a measure of the actual capacities for treating patients with severe courses of COVID-19. Information on free intensive care units was missing for four NUTS-3 regions from different NUTS-2 regions. In these NUTS-2 regions, free intensive care units were related to the sum of population of all NUTS-3 regions with complete data for intensive care units.

Additionally, we controlled for several other regional characteristics in order to isolate health infrastructure effects from other sources of regional variation. We accounted for the share of people receiving social assistance for households with long-term unemployed members (SGB-II) in 2020 and the gross domestic product (GDP) in 2019 as indicators for the regional economic situation. Finally, we included the population density in 2020 at the place of residence. We used the most recent available data for each indicator. Table 1 provides further information on the operationalization and the coding of the variables.

**Table 1:** Operationalization and coding of the independent variables

**Individual characteristics**	
Gender	0 = male, 1 = female
Age	0 = 18–29 years, 1 = 30–49 years, 2 = 50–69 years, 3 = 70 years and older
Migrant background	0 = no, 1 = yes (direct or indirect migration (2nd generation)
Educational level	0 = low, 1 = medium, 2 = high, measured by the CASMIN-classification
Household net income	0 = lower 25%, 1 = middle 50%, 2 = upper 25%, equivalised ($income/\sqrt{size\ of\ household}$)
Chronic illness	0 = no, 1 = yes, at least one
Subjective health	0 = very good, 1 = good, 2 = satisfactory, 3 = poor, 4 = bad
COVID-19 as a threat for one’s own health	How likely do you think it is that the novel coronavirus will cause you to become critically ill in the next 12 months? 0–33%: low, 34–66%: medium, 67–100%: high
Health insurance	0 = mandatory health insurance, 1 = private health insurance
**Regional characteristics (NUTS-2 level)**	
COVID-19 infections	Number of infections per 1000 inhabitants from January to February 2021
Hospital beds	Number of hospital beds per 1000 inhabitants in 2019
Intensive care units	Average of daily free intensive care beds per 100,000 inhabitants from January to February 2021
GDP	EUR per capita in 2019
Poverty rate	Share of recipients of social assistance for households with long-term unemployed household member (SGB-II) in 2020
Population density	Average inhabitants per km^2^ in 2020

### Statistical modelling

We apply multilevel logistic random-intercept regression to account for the clustering of the individual level survey data at NUTS-2 level. For a set of explanatory variables, we estimated odds ratios (OR) for the likelihood to be “somewhat” or “very” concerned to receive the needed medical treatment in case of an infection (the dependent variable). The model is a random-intercept model with individual-level (level 1) and regional-level (level 2) variables which are aggregated across time for daily data [60]. Prior multilevel analyses of macro-level influences on wellbeing typically operate with country data, but care infrastructure varies a lot by region and the reachability of services is what matters for individual wellbeing [see also 41]. Analyses were performed with Stata V.16.

## Results

### Description

Table 2 shows percentages on our variables of interest. A substantial part of the sample reported worries about inadequate COVID-19-treatment: 41% reported to be very or somewhat concerned about not receiving adequate treatment in case of a COVID-19 infection. Applying survey weights to account for varying sample and response probabilities of SOEP respondents changes the estimated percentage of people to be very/somewhat worried only slightly to 42% (95%-CI: 40–44%). Around two thirds of the sample were women, the majority was aged 50 years and older (mean 55.4; range 20 to 100), and around 16% had a migrant background. With respect to socioeconomic conditions, the majority of the sample had a medium or even high educational level (42.9%, respective 32.2%), whereas the proportion of individuals with low educational level was 24.9%. As defined beforehand, around one quarter of the sample belonged to the lowest income group, 50% belonged to the middle-income group and another 25% to the highest income group. A substantial part of the sample reported to suffer from at least one chronic illness (40.5%). While the majority rated their overall health as good (45.5%) or even very good (14.7%), 73.1% considered COVID-19 as a threat for their own health in case of an infection. Most respondents (85.5%) had a mandatory health insurance, while 14.5% had a private health insurance.

**Table 2:** Sample description (*n* = 5045)

**Variable**	
*Individual characteristics*	% (n)
Worries	
Not concerned	59.5 (3001)
Very or somewhat concerned	40.5 (2044)
Gender	
Male	39.1 (1971)
Female	60.9 (3074)
Age	
Under 30 years	5.4 (272)
30–49 years	30.3 (1527)
50–69 years	43.6 (2197)
70 years and older	20.8 (1049)
Migrant background	
No	84.2 (4247)
Yes	15.8 (798)
Educational level	
Low	24.9 (1258)
Medium	42.9 (2164)
High	32.2 (1623)
Household income	
Lower 25%	1097 (mean) (1235)
Middle 50%	2042 (mean) (2546)
Upper 25%	3834 (mean) (1264)
Chronic illness	
No	59.5 (3004)
Yes	40.5 (2041)
Subjective Health	
Very good	14.7 (743)
Good	45.5 (2295)
Satisfactory	29.4 (1481)
Poor	8.6 (435)
Bad	1.8 (91)
COVID-19 as a threat for one’s own health	
Low	73.1 (3688)
Medium	24.2 (1221)
High	2.7 (136)
Health insurance	
Mandatory health insurance	85.5 (4312)
Private health insurance	14.5 (733)
*Regional characteristics*	Mean (min. – max.), SD
COVID-19 infections per 1000 inhabitants	0.861 (0.478–1.546), SD = 260.850
Hospital beds per 1000 inhabitants	6.1 (3.8–7.5), SD = 0.839
Free intensive care units per 100,000 inhabitants	6.0 (3.2–13.0), SD = 1.849
GDP	39,884.0 (28,993.0 – 67,017.0), SD = 9390.462
Poverty rate	6.4 (2.6–14.9), SD = 2.770
Population density	453.8 (69.1–4114.81), SD = 840.324

Source: Own calculations based on SOEP-Cov, Statistische Ämter des Bundes und der Länder (2022), infas 360 GmbH (2021a, 2021b)

Regarding the regional characteristics, the average COVID-19 infections throughout the survey period was 0,816 per 1000 inhabitants. The average number of hospital beds per 100,000 inhabitants was 6.1, and the number of free intensive care units per 10,000 inhabitants amounted to 6.0 at the day of the interview. Regarding the remaining variables at the regional level, the average GDP was 39,884.0 EUR per capita and the average poverty rate amounted to 6.4%. The average population density was at 453.8 inhabitants per km^2^.

**Fig. 1:**
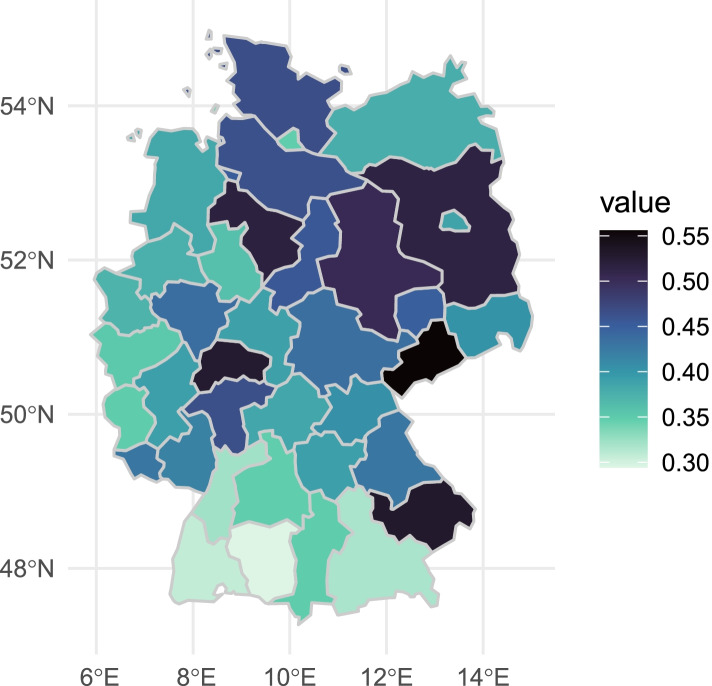
Regional inequalities (NUTS-2) in worries about inadequate COVID-19-treatment (*n* = 5045). Source: Own calculations based on SOEP-Cov. Minimum: 0.31 (DE13, Freiburg), Maximum: 0.56 (DED4, Chemnitz)

Worries about inadequate COVID-19-treatment were unevenly distributed across German regions. Figure 1 shows the percentage of respondents who were somewhat or very worried about not receiving the needed medical treatment in case of a COVID-19 infection. Darker areas reflect a higher share of worried respondents. There are pronounced regional inequalities with a higher share of respondents reporting worries in the Northern and Eastern part of Germany as compared to the Western and Southern regions, and less worries in densely populated areas.

### Regression analysis

Table 3 shows the OR for worries about inadequate COVID-19-treatment based on multilevel logistic regression models. Model 1 includes all confounders on the individual level, in model 2, the macro indicators of NUTS-2 regions are included.

**Table 3:** Determinants of worries about inadequate COVID-19 treatment (*n* = 5045)

	**Model 1**		**Model 2**	
	**OR**	**95% CI**	**OR**	**95% CI**
*Individual characteristics*				
Ref: Male				
Female	1.351***	1.193–1.530	1.352***	1.194–1.531
Ref: Under 30 years				
30–49 years	1.733***	1.283–2.342	1.727***	1.279–2.333
50–69 years	1.860***	1.382–2.503	1.851***	1.375–2.492
70 years and older	1.878***	1.367–2.581	1.879***	1.367–2.581
Ref: No migrant background				
Migrant background	1.326**	1.118–1.573	1.339**	1.129–1.589
Ref: Low educational level				
Medium	0.786**	0.674–0.916	0.775***	0.665–0.903
High	0.719***	0.603–0.858	0.704***	0.590–0.840
Ref: Lower 25% household income				
Middle 50%	0.873	0.753–1.012	0.888	0.765–1.030
Upper 25%	0.752**	0.621–0.910	0.769**	0.635–0.931
Ref: No chronic illness				
Chronic illness	1.166*	1.022–1.330	1.155*	1.013–1.318
Ref: Very good subjective health				
Good	1.482***	1.226–1.790	1.480***	1.225–1.789
Satisfactory	1.903***	1.551–2.333	1.895***	1.545–2.324
Poor	2.069***	1.588–2.696	2.061***	1.581–2.685
Bad	1.725*	1.084–2.743	1.701*	1.069–2.706
Ref: COVID-19 as a threat for own health: Low				
Medium	1.878***	1.639–2.152	1.862***	1.625–2.133
High	2.926***	2.019–4.242	2.907***	2.006–4.214
Ref: Mandatory health insurance				
Private health insurance	0.864	0.713–1.048	0.877	0.723–1.063
*Regional characteristics*				
COVID-19 infections			1.001***	1.0002–1.001
Hospital beds			0.889*	0.792–0.999
Free intensive care units			0.961	0.918–1.007
GDP per capita			1.000	1.000–1.000
Poverty rate			1.073**	1.026–1.122
Population density			1.000*	1.000–1.000
Log Likelihood	3221.955		3212.856	
Number of NUTS-2 regions	38		38	
Number of respondents	5045		5045	

Source: Own calculations based on SOEP-Cov, Statistische Ämter des Bundes und der Länder (2022), infas 360 (2021a, 2021b). * *p* < 0.05, ** *p* < 0.01, *** *p* < 0.001

In the full model, Women (OR 1.35, 95%-CI = 1.19–1.53), older respondents (OR 1.73, 95%-CI = 1.28–2.33 for age group 30–49 years; OR 1.85, 95%-CI = 1.38–2.49 for age group 50–69 years; OR 1.88, 95%-CI = 1.37–2.58 for age group 69+ years) and persons with migrant background (OR 1.33, 95%-CI =1.13–1.59) were more likely to report to be concerned. Inequalities in worries were also evident with regard to SES: respondents with medium and high educational level (OR 0.78, 95%-CI = 0.67–0.90 for medium educational level; OR 0.70, 95%-CI = 0.59–0.84 for high educational level) and high income (OR 0.77, 95%-CI = 0.64–0.93) were less likely to report concerns about inadequate treatment. Moreover, health status was relevant as expected. Respondents with chronic illnesses (OR 1.16, 95%-CI = 1.01–1.32), lower subjective health (OR between 1.48 and 2.06) and those who consider COVID-19 as threat for their individual health (OR 1.86, 95%-CI = 1.63–2.13 for medium threat, OR 2.91, 95%-CI = 2.01–4.21 for high threat) were more likely to report worries about inadequate COVID-19-treatment. Regarding type of health insurance, privately insured respondents do not differ significantly from respondents with mandatory health insurance.

Besides individual characteristics, also regional characteristics were relevant. A higher regional number of COVID-19 infections was associated to worries of inadequate medical treatment in case of contracting the virus (OR 1.001, 95%-CI = 1.0002–1.001), whereas a higher number of hospital beds was linked to a decreased chance for reporting worries (0.89, 95%-CI =0.79–0.99). In contrast, the number of free intensive care units was not associated with worries about inadequate treatment. Moreover, also the poverty rate at the regional level was linked to an increased chance (OR 1.073, 95%-CI =1.026–1.122) for reporting worries about inadequate treatment.

## Discussion

This is the first study to investigate the determinants of worries about inadequate treatment in case of a COVID-19 infection in a regional context based on a representative sample of the German population. The analysis considers social inequalities at the individual and regional level, as well as the regional spread of COVID-19 and healthcare infrastructure. Regarding individual-level characteristics, our analysis revealed pronounced social inequalities in the expected directions: Women, older respondents and persons with migrant background were more likely to be concerned not to receive an adequate medical treatment in case of a COVID-19 infection.

Moreover, socioeconomically disadvantaged respondents with lower income and education were more concerned. This could be due to inadequate knowledge about COVID-19 [30, 32] [Arcadio et al. 2021, De Kock et al. 2021], but might also reflect that they accurately reflect that people of lower socioeconomic status are more likely to get infected, to be severely ill and to die from COVID-19 [61–64]. Since people tend to have social relationships with others who are similar to themselves [65], socially disadvantaged respondents are more likely to have experienced severe cases of or deaths from COVID-19. Furthermore, they might have worse experience with the health care system per se due to lower financial and information resources [66]. Accordingly, they may be more likely to see COVID-19 and inadequate treatment as a threat.

A lower health status was related to worries about inadequate medical treatment which is in line with previous findings [1, 23, 67]. However, it did not matter whether somebody had a private insurance or not – which hints to the fact that worries were more fundamental than “just” being able to finance the right treatment, presumably as treatments were still very limited or not yet available.

Besides individual characteristics, also regional inequalities were evident. Worries about inadequate treatment were more common in regions with worse health infrastructure represented by number of hospital beds, but are not related to the number of free intensive care units. The relation of both health infrastructure indicators to concerns might differ because respondents are more familiar with health infrastructure represented by hospital beds available than they are with the number of currently free intensive care units. Whereas many respondents might have been on normal ward as patient or visitor, probably only few have this experience with intensive care. Therefore, intensive care units might not reflect perceived health infrastructure in a region, although they are crucial for treatment of severe cases of COVID-19 infections. Furthermore, also when controlling for the individual socioeconomic position, regional poverty rates were linked to more individual worries about inadequate medical treatment.

All this provides further hints to a reinforcement of existing health inequalities during the pandemic – which indeed seems to be an accelerator for social inequalities in many dimensions, and a burning glass for structural inequalities within and between regions, as has been claimed already [68]. This study also shows that there are living conditions and contextual circumstances which can prevent or at least reduce fears of inadequate medical treatment in case of COVID-19 infection – and such should be invested in, not only to prevent fears, but also ensure actual adequate medical treatment, especially during pandemic times.

### Limitations

This observational study is not without limitations. While our findings that regional health infrastructure is related to worries about inadequate medical treatment matches findings from earlier studies on health infrastructure and wellbeing [40, 41], we can not make causal claims. Other unobserved characteristics at the regional or individual level can explain observed relationships, too. In the beginning of 2021, regional variation in concerns could for example also be a product of different regional predominant political orientations across Germany [69] which we cannot measure and control for adequately.

Moreover, the question remains open if there are systematic differences in the understanding of the phrasing “receive the necessary medical treatment if you contract the coronavirus” among groups of respondents. Apart from the fact, that respondents might perceive differences in the medical treatment they need in case of a COVID-19 infection (e.g., based on their health status), it might be hard for individuals to judge which medical treatment is adequate for them in the hypothetical situation of an infection.

## Conclusions

Nevertheless, our study is an important contribution to previous research on determinants of mental wellbeing in light of the ongoing pandemic. Based on a representative sample of the German population, the analysis not only indicates that several social groups are confronted with more worries about inadequate medical treatment in case of a COVID-19 infection, but also highlights the need for considering regional-level influences, such as the spread of the virus, as well as the availability of health care infrastructure. Besides individual socioeconomic status, regional social inequalities seem to be linked to individual worries [70] – also in the case of COVID. The analysis provides several starting points for future studies on different contextual measures impacting on various dimensions of (social) health inequalities. In order to get closer to the underlying mechanisms, public health research infrastructures should not only invest in comparable and fine-grained regional health care and social indicators, but also in their individual perception. As we showed, it is not the objective facts alone but also the (differential) subjective awareness of citizens which is linked to wellbeing. This may be especially true during pandemic times and crises where knowledge and opinion are hard to distinguish. Intersectional inequalities as well as their perception should be considered in any public health measure to ensure wellbeing of all citizens.

## Data Availability

The data are available for scientific purposes after signing a data distribution contract with DIW Berlin (https://www.diw.de/en/diw_01.c.601584.en/data_access.html). The analyses during the current study are available from the corresponding author upon request.
